# Supplementary figures and images for: Nutrient sensing pathways regulating adult reproductive diapause in C. elegans
Source: PLoS One. 2022 Sep 16;17(9):e0274076. doi: 10.1371/journal.pone.0274076 (PMC9480990; doi:10.1371/journal.pone.0274076)

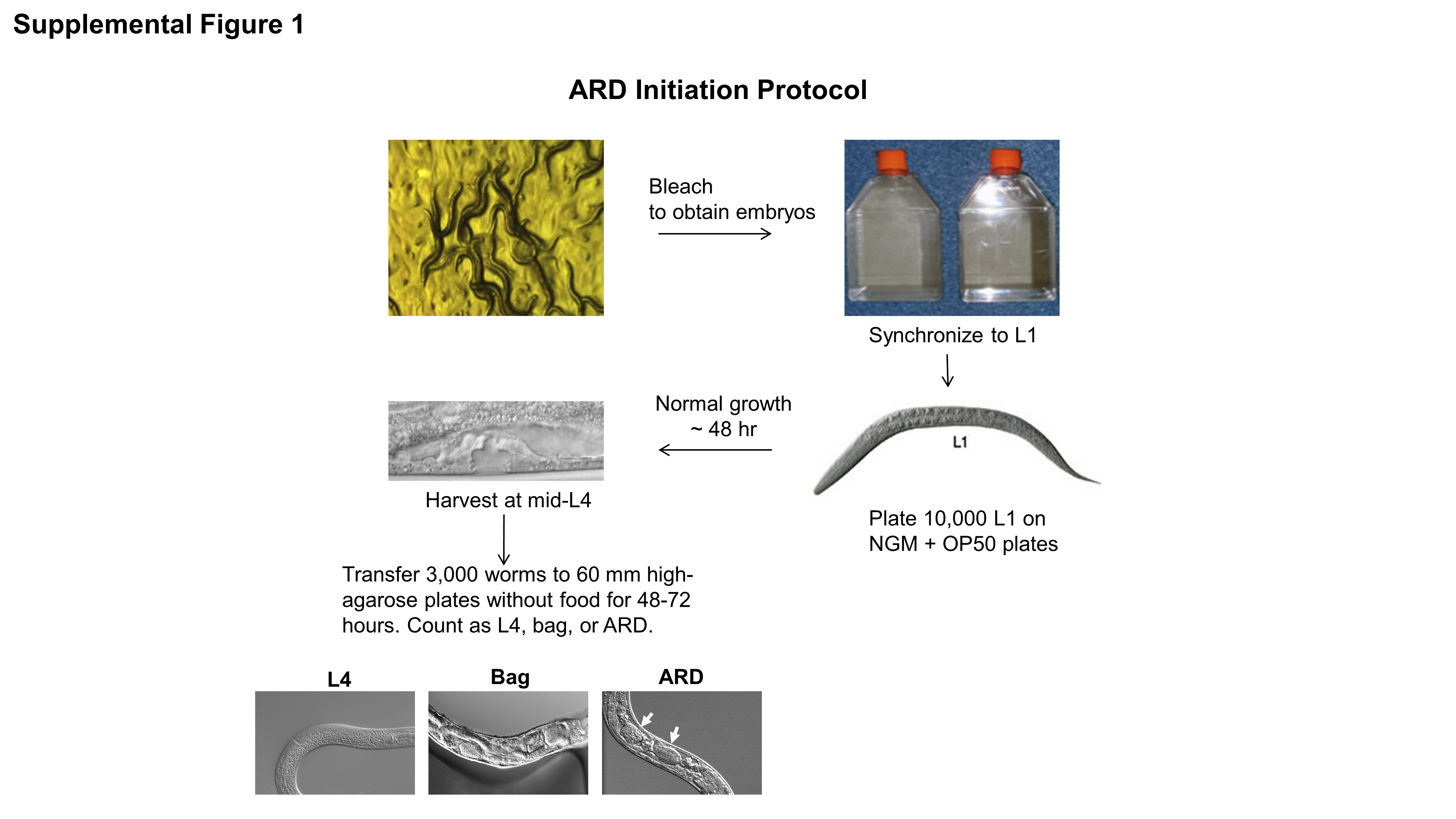

Supplement: S1 Fig — As described in Materials and Methods, worms were bleached to obtain synchronized L1 larvae. The L1s were then plated at a density of 10,000 per plate, harvested at mid-L4 stage, and grown on plates without food for 48–72 hours. At this stage worms were counted to determine the percentage of animals in arrested L4, bagged adults, or ARD (arrows indicate retained embryos) as shown in the representative photos. (TIF) [file pone.0274076.s001.tif]

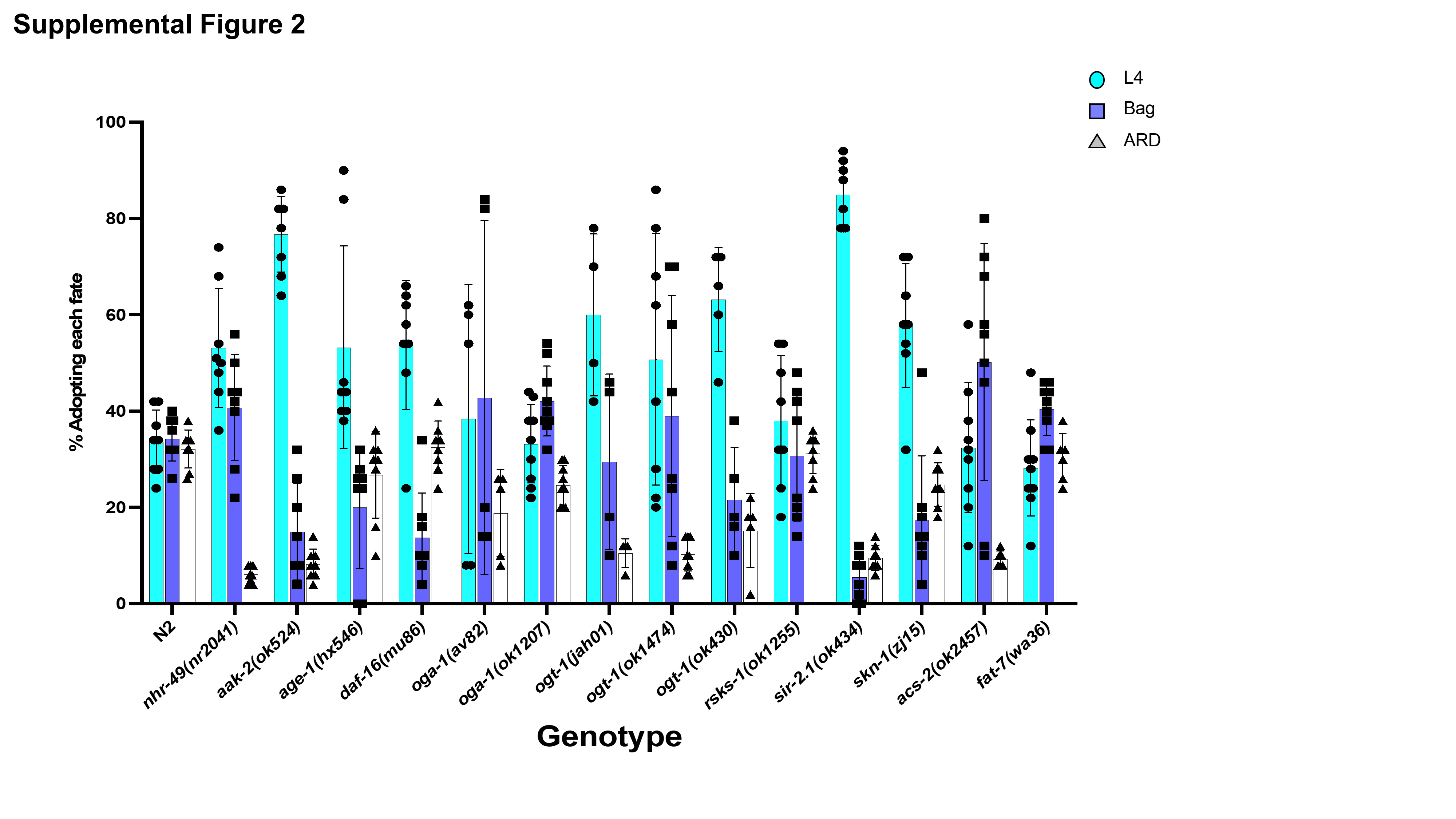

Supplement: S2 Fig — Percent of worms from each respective genotype in L4 (blue bar), bag (purple bar) or ARD (grey bar) are represented. The L4 and bagging fates tended to have a higher degree of variability than ARD entry, as evidenced by a higher standard deviations. Select strains had a marked increase in the percentage of worms in L4 including aak-2(ok524), age-1(hx546), daf-16(mu86), sir-2.1(ok434), and skn-1(zj15). Other mutant strains, nhr-49(nr2041), oga-1(ok1207), and ogt-1(jah01), were more similar to wildtype in terms of having a more even distribution between L4 and bagging even though this set was defective for ARD entry. (TIF) [file pone.0274076.s002.tif]

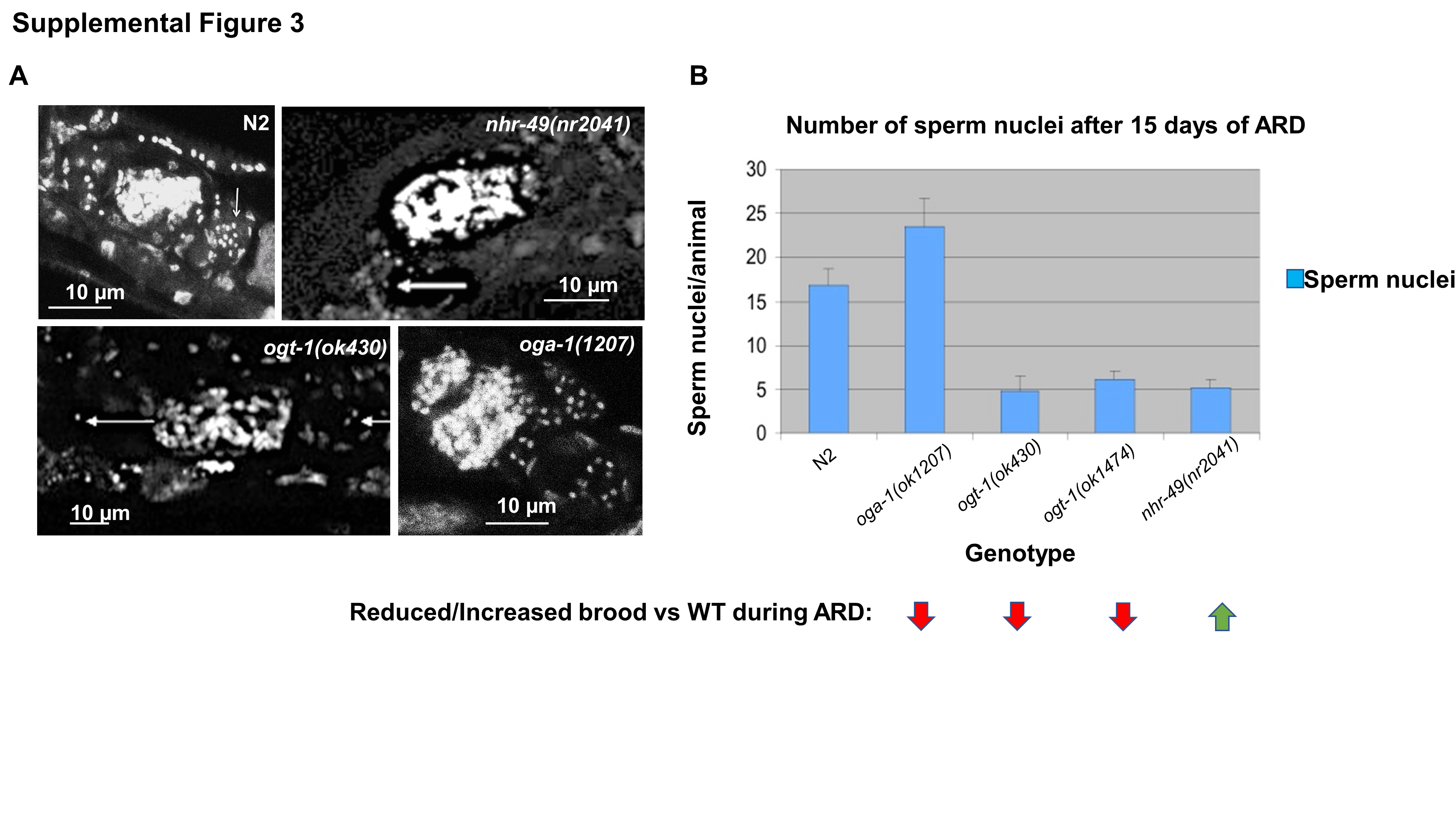

Supplement: S3 Fig — (A) DAPI staining of worms after 15 days in ARD reveals fewer sperm in the spermatheca of ogt-1(1474), and nhr-49(nr2041) compared to wild-type N2 worms. In the oga-1(ok1207) worms, more sperm were observed. White arrows indicate examples of sperm nuclei. (B) Whereas most of the strains analyzed saw a decrease in the number of sperm nuclei present at day 30 of ARD, oga-1(ok1207) had an overall increase compared to wildtype. However, these dynamics did not correlate with changes in brood size among selfed individuals. Green arrows indicate increased brood numbers vs wildtype and red arrows indicate decreased brood vs wildtype. (TIF) [file pone.0274076.s003.tif]

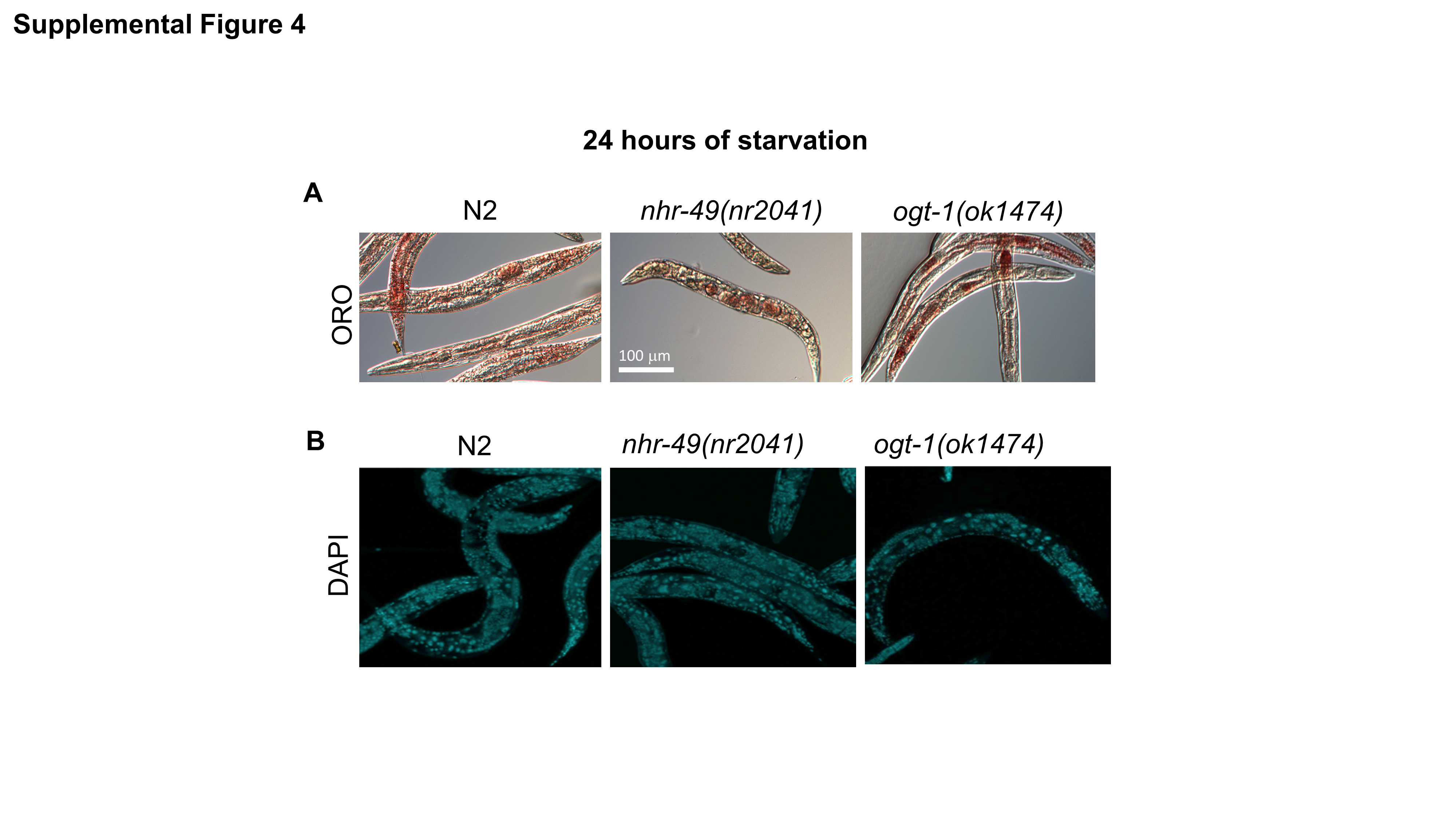

Supplement: S4 Fig — (A) Decrease in ORO signal were observable at 24 hr after worms were placed on ARD plates (as compared to control fed worms in Fig 5), as shown with representative strains. (B) Strains were stained with DAPI to demonstrate efficient small-molecule penetrance of cuticles across strains, indicating that changes in ORO staining are not related to differences in cuticle penetrance between strains. (TIF) [file pone.0274076.s004.tif]

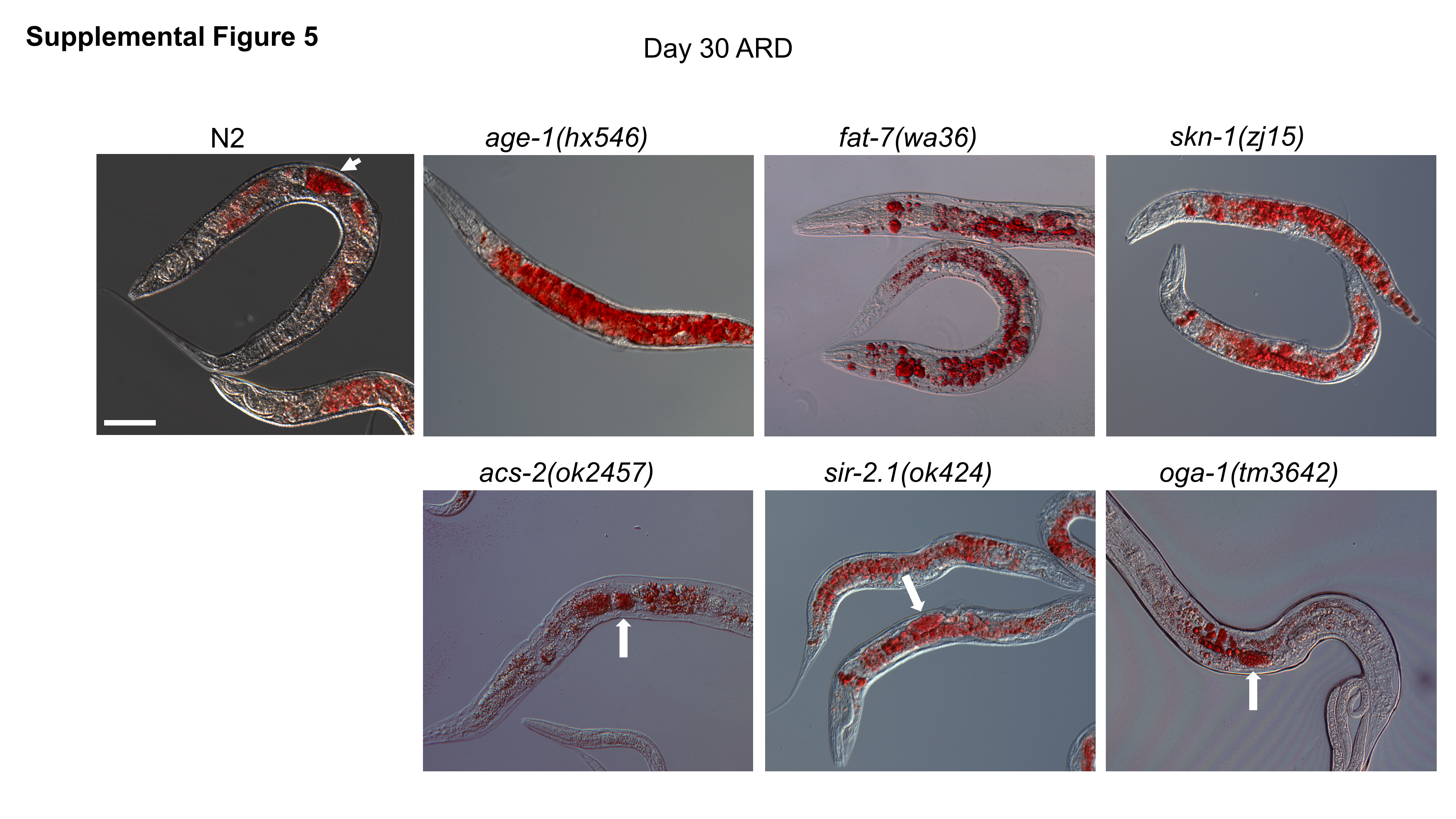

Supplement: S5 Fig — In addition to the ogt-1-dependent pathway, we also looked at strains with diverse phenotypes to see if changes were unique to the ogt-1 pathway. We observed that outside of the ogt-1 pathway, strains that did not influence either entry or exit (age-1(hx546)), strains that also only influenced entry (sir-2.1(ok424)), and strains that influenced only recovery (skn-1(zj15)) had a marked increase in ORO staining/TAG stores compared to both wildtype and the ogt-1 pathway. We also observed this same pattern with genes downstream of nhr-49, such that acs-2 (with a defective ARD entry) had reduced ORO staining but fat-7 (no defect in ARD entry) had a stronger ORO signal. Arrows indicate retained embryos, which show high ORO staining. For easier comparison to control, the image of N2 worms in ARD from Fig 5 is included here. (TIF) [file pone.0274076.s005.tif]

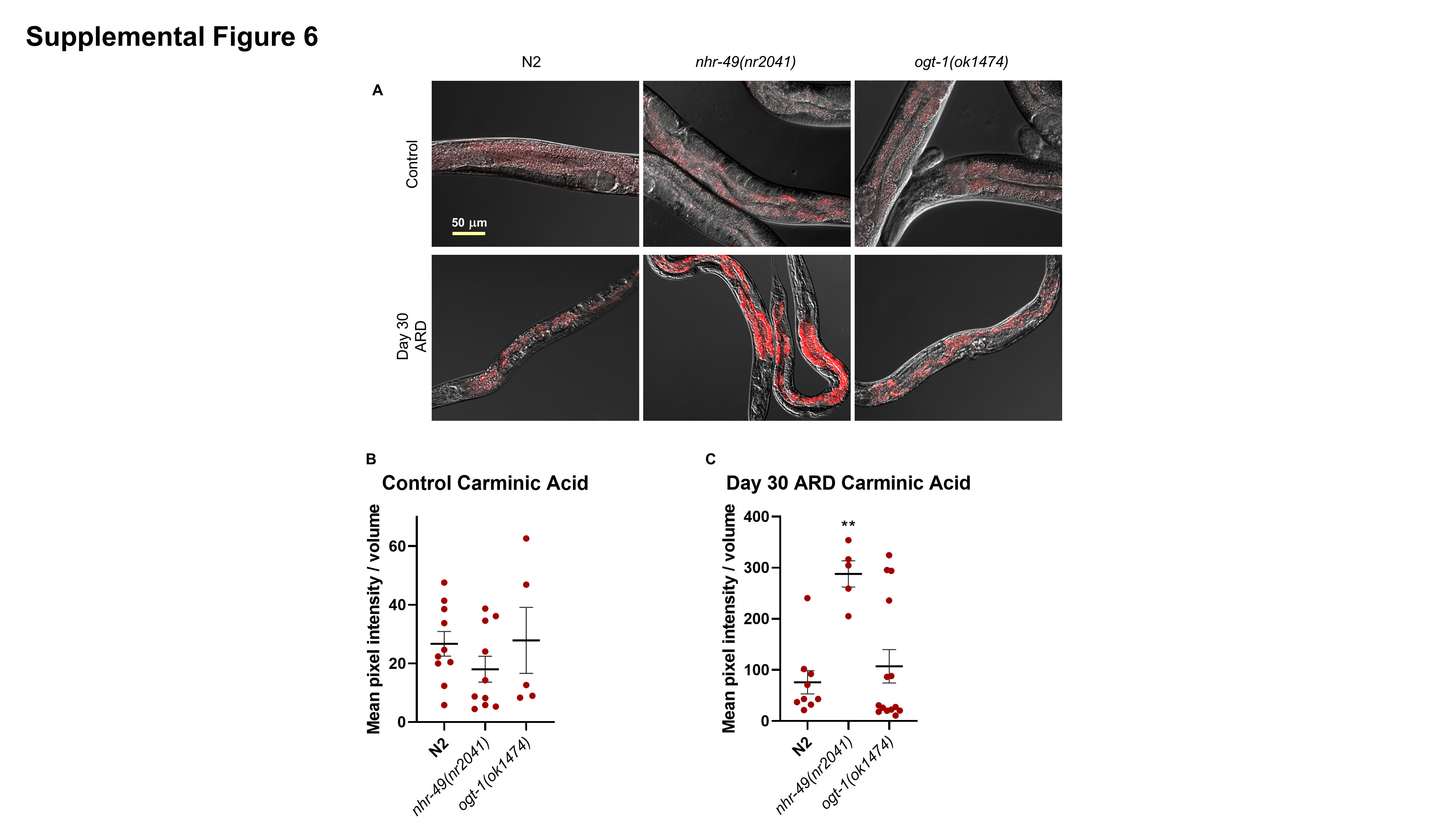

Supplement: S6 Fig — (A) Carminic acid staining (indicative of glycogen and trehalose levels) varied greatly between strains. As we have previously reported [22], ogt-1(1474) showed slightly higher levels of carminic acid staining than other strains in standard husbandry conditions, though in this study this change did not reach significance. After 30 days of ARD (lower panels) we noted that nhr-49(nr2041) had a dramatic increase in staining, while wild type and ogt-1(ok1474) did not. These results did not correlate with the observed defect in ARD entry for these strains. (B) ImageJ based quantification of carminic acid fluorescence by pixel intensity. P-value **** = <0.0001, as determined by two-way ANOVA. (TIF) [file pone.0274076.s006.tif]
