# Supplementary material for: Nutrient sensing pathways regulating adult reproductive diapause in C. elegans
Source: PLoS One. 2022 Sep 16;17(9):e0274076. doi: 10.1371/journal.pone.0274076 (PMC9480990; doi:10.1371/journal.pone.0274076)
Supplement: S1 Table — The table shows the average brood size under normal husbandry (control) or re-fed animals following 5 or 10 days in ARD. A high degree of variability was observed between and within experimental replicates for the average brood size (SEM). These brood sizes did not correlate to fate adoption of each strain nor with influence on any of stages of ARD. Red indicates a decrease compared to wildtype whereas green indicates an increase vs wildtype. (PDF) [file pone.0274076.s007.pdf]

Supplemental Table 1

Genotype	Average brood size			SEM		% brood vs control	
	Control	Day 5	Day 10	Day 5	Day 10	Day 5	Day 10
**N2**	243.9	50.6	27.9	6.6	6.7	20.7	11.4
nhr-49(nr2041)	157.1	105.8	33.5	8.4	5.7	67.3	21.3
aak-2(ok524)	210.8	33.5	12.0	5.3	4.0	15.9	5.7
age-1(hx546)	109.3	50.8	21.3	9.4	4.9	46.5	19.5
daf-16(mu86)	185.3	34.8	2.8	5.3	2.0	18.8	1.5
oga-1(av82)	214.6	68.3	20.0	7.3	5.6	31.8	9.3
oga-1(ok1207)	208.6	24.0	8.8	3.2	2.8	11.5	4.2
ogt-1(jah01)	209.2	88.5	10.3	16.9	2.5	42.3	4.9
ogt-1(ok1474)	151.4	46.4	8.8	6.0	1.6	30.7	5.8
ogt-1(ok430)	194.6	31.7	8.7	9.8	2.1	16.3	4.5
rsks-1(ok1255)	114.4	72.1	19.2	19.5	4.6	63.0	16.8
sir-2.1(ok434)	87.1	51.5	21.3	6.8	5.0	59.1	24.5
skn-1(zj15)	116.5	26.7	1.3	9.8	0.8	22.9	1.1
