# Supplementary material for: Nutrient sensing pathways regulating adult reproductive diapause in C. elegans
Source: PLoS One. 2022 Sep 16;17(9):e0274076. doi: 10.1371/journal.pone.0274076 (PMC9480990; doi:10.1371/journal.pone.0274076)
Supplement: S1 Methods — Detailed description of image analysis and quantification of images of Oil Red O stained C. elegans adults. (PDF) [file pone.0274076.s008.pdf]

Supplementary Methods

Image Analysis

When quantifying % differences in the amount of a substance S in a microscope image of biological cells, the process is relatively easy when S is labeled with a fluorescent probe (F), such as when carminic acid (acting as F) labels stored glycogen (acting as S). The labeling can be performed such that a constant stoichiometric relationship exists between F and S, so that the digitized intensity (I) of light (collected photons) from F in each pixel is proportional to the concentration (C) of substance S in the volume represented by that pixel:

$$I \propto F \propto C \qquad \text{(Eq1)}$$

or working backwards from the measurement for I:

$$C \propto F \propto I \qquad \text{(Eq2)}$$

Thus, in the case of fluorescence, one can easily measure % differences in C (or total amount) simply by measuring % differences in I. This relationship is usually assumed by default when interpreting fluorescence microscopy images, as was done in this paper when quantitating carminic acid.

Although it is possible to quantitate ORO using its fluorescence—a few examples can be found in the literature-- there are advantages to using absorbance instead of fluorescence: 1) avoiding competition with autofluorescence when recording the ORO signal; and 2) transmitted signal typically dwarfs the signal from fluorescence, which typically means less photodamage, and better overall signal-to-noise ratio (SNR). The disadvantage is that relating pixel intensities to concentration or amount of a substance requires more work.

The Beer–Lambert–Bouguer Law (1) has been used extensively in other (non-imaging) instrumentation to determine concentrations/amounts of substances, e.g. spectrophotometric determination of DNA concentration using absorbance at 260 nm:

$$\varepsilon \ell C = A = \log (I_o / I) \qquad \text{(Beer–Lambert–Bouguer Law)}$$

or for practical difference measurements:

$$C \propto A \propto \log (I_o / I) \qquad \text{(Eq3)}$$

…where A is absorbance of an absorbent probe for S with constant stoichiometry between them; ε is the molar absorption coefficient of the substance; ℓ is the path length traveled through the substance; 'I' is the measured intensity after passing through the sample; and I_o, often called the "incident" light intensity in the literature, is more accurately the intensity that the light would have if there were no substance S in the light path.

When applying the Beer–Lambert–Bouguer Law to images from a transmitted light microscope of a live sample, determination of I_o for each pixel is problematic. Using a "blank" as background for all pixels in the image, like one would in a DNA spectrophotometer, is not applicable—even without substance S, cells diffract and absorb light in a heterogenous way, or we would not be able to see them in transmitted light, and record them as images with pixels at different intensities. Each pixel, representing a unique light path through the sample, has a blank associated with it. Ideally, a true blank would be found for each pixel by imaging the same sample minus the ORO, which is practically impossible.

The trick is to take advantage of the high dependence of ORO's absorbance (ε from the equation above) on wavelength: measure 'I' at a wavelength ($\lambda 1$) that absorbs very highly; and measure 'I_o' at a wavelength ($\lambda 2$) with much lower absorption, using the latter as the "blank" measurement:

$$C \alpha \ A \alpha \ \log (I_o / I) \ \alpha \ \log (I_{\lambda 2} / I_{\lambda 1}) \qquad \text{(Eq4)}$$

If the relation in Eq4 were valid, standard ratio imaging techniques could be used to measure differences in ORO concentrations, hence TAG concentrations/amounts. Although it seems intuitive to substitute $I_{\lambda 2}$ for I_o when measuring relative differences in A and C, a more rigorous derivation is called for.

Consider the absorbance values at the two wavelengths separately, hypothesizing virtual values for the blanks, $I_{o,\lambda 1}$ and $I_{o,\lambda 2}$:

$$A_{\lambda 2} = \log (I_{o,\lambda 2} / I_{\lambda 2}) = \log (I_{o,\lambda 2}) - \log (I_{\lambda 2}) \qquad \text{(Eq5)}$$

$$A_{\lambda 1} = \log (I_{o,\lambda 1} / I_{\lambda 1}) = \log (I_{o,\lambda 1}) - \log (I_{\lambda 1}) \qquad \text{(Eq6)}$$

Assumption #1: The relative absorbance between wavelengths λ1 and λ2 is constant for all practical purposes; i.e. $A_{\lambda 1} = k * A_{\lambda 2}$, where k>>1 is a proportionality constant.

$$A_{\lambda 1} = k * A_{\lambda 2} \ , \ k >> 1 \qquad \text{(Eq7)}$$

The absorption spectra of ORO in various hydrophobic solutions (Adapted from (2)) is shown below, demonstrating a wide range in the absorbance over the range 400-700nm, the range where instrumentation laser sources are normally found. We assume that the molecular environment in *c. elegans* where ORO binds in any appreciable quantity will produce a similar, but constant shape for the absorbance curve. Laser wavelengths commonly found on confocal microscopes are shown overlaid on the graph. The laser line 639nm seems like the best choice for $\lambda 2$, but further assumptions will help make the choice for us.

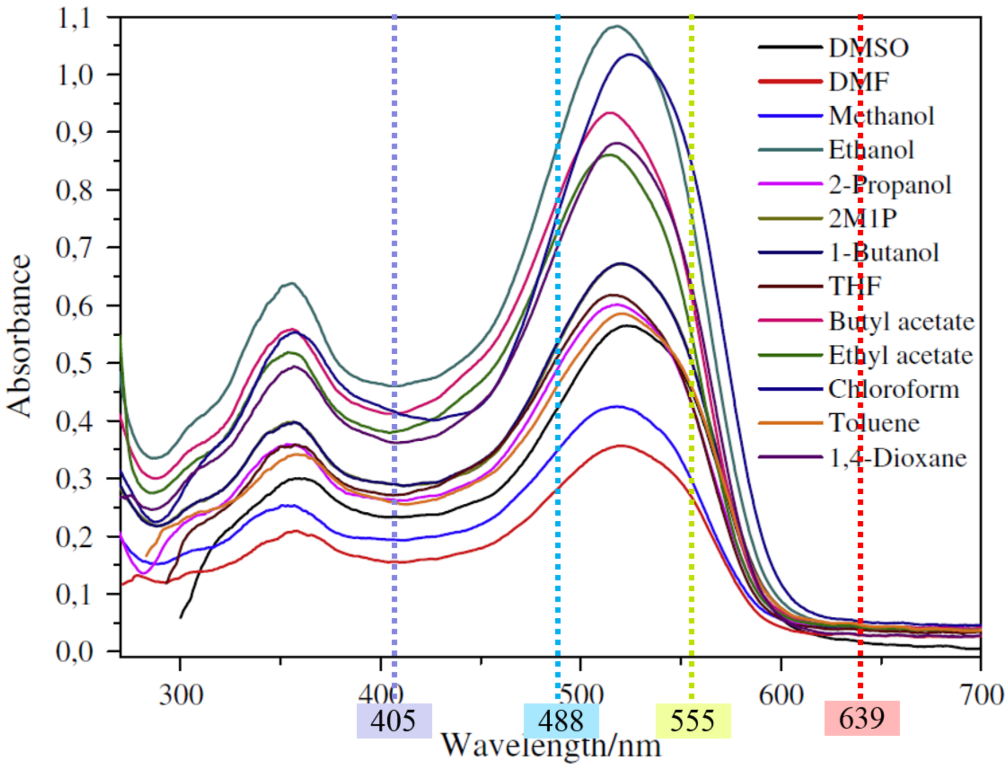

Absorption spectra of ORO at room temperature.

Adapted from Sidir et al. (2015)

We can use Eq7 with Eq5 and Eq6 to yield Eq8:

$$A_{\lambda 1} \quad = \quad k * \log (I_{o,\lambda 2} \ / \ I_{\lambda 2}) \qquad \text{(Eq8)}$$

Assumption #2: The inherent optical differences between the two images acquired at λ1 and λ2 are negligible, i.e. the ratio λ1/ λ2 should be as close to 1 as possible.

$$\lambda 1 / \lambda 2 \sim 1 \qquad \text{(Eq9)}$$

We can't forget that image formation through refractive optics depends on wavelength, and these wavelength-dependent differences need to be minimized. Otherwise, the effective path length would not be the same for λ1 and λ2, and any assumptions that allow the two images to be combined on a pixel-by-pixel basis are not valid. The optics of the microscope should be (and are, for this paper) color-corrected for λ1 and λ2.

From the laser choices shown on the graph above, 555nm and 639nm seem to be the best choices for λ1 and λ2, respectively, to keep the ratio close to 1 while maximizing the ratio k.

Assumption #3: The ratio of the virtual blank pixel intensities, m= $I_{o,\lambda 2}$ / $I_{o,\lambda 1}$, can be normalized to m=1; i.e. the image needs to be "color-balanced" after acquisition. Normally, since the ratio of illumination intensities will be a constant, the ratio of the virtual blanks will also be some constant value (m). Not only does this normalization, done on an image-by-image basis, make the images more visually appealing, it normalizes for subtle differences when initially setting up the channels, as well as for drifts over time in the output of the different laser sources for λ1 and λ2. A welcome consequence is that the math becomes easier.

$I_{o,\lambda 1} = m * I_{o,\lambda 2}$ becomes:

$$I_{o,\lambda 1} = I_{o,\lambda 2} \quad \text{(Eq10)}$$

Now substitute into Eq8:

$$A_{\lambda 1} = k * \log (I_{o,\lambda 1} / I_{\lambda 2}) = k * [\log (I_{o,\lambda 1}) - \log (I_{\lambda 2})] \quad \text{(Eq11)}$$

Rearranging Eq6 to substitute for $\log (I_{o,\lambda 1})$ in Eq11:

$$A_{\lambda 1} = k * \log (I_{o,\lambda 1} / I_{\lambda 2}) = k * [(A_{\lambda 1} + \log (I_{\lambda 1})) - \log (I_{\lambda 2})] \quad \text{(Eq12)}$$

Rearranging/solving for $A_{\lambda 1}$:

$$A_{\lambda 1} = (k/(k-1)) * \log (I_{\lambda 2} / I_{\lambda 1}) \quad \text{(Eq13)}$$

This last equation shows absorbance as a function of the ratio of the two images, with a constant k-based term. The k-term is eliminated if we compare relative differences in absorbance, in which case we write it more generally as:

$$A \ \alpha \ \log (I_{\lambda 2} / I_{\lambda 1}) \quad \text{(Eq14)}$$

Combining Eq14 with the Beer–Lambert–Bouguer Law, we arrive at our hoped-for destination, Eq4:

$$C \ \alpha \ A \ \alpha \ \log (I_{\lambda 2} / I_{\lambda 1}) \quad \text{(Eq4)}$$

An easy way of testing if the Assumptions for Eq12 are valid is by performing the same quantitative analysis on samples that do not have ORO in them but are otherwise equivalent to the measured samples. If there is negligible measured absorbance $A_{\lambda 1}$ as when compared to samples with ORO, then the technique can be considered robust for that particular sample, probe, and optics.

Finally, we note that standard background subtraction protocol for ratio imaging should be followed. Either the black level of the instrument is zeroed out before measurement (as we did for this paper), or this background is subtracted post-acquisition, prior to ratioing any pixel intensities.

References

1. Harris DC. Quantitative Chemical Analysis. 6 ed. New York: W.H. Freeman and Company; 2003.
2. Sıdır İ, Gülseven Sıdır Y. Estimation of ground and excited state dipole moments of Oil Red O by solvatochromic shift methods. Spectrochim Acta A Mol Biomol Spectrosc. 2015;135:560-7.
