# Supplementary material for: Nutrient sensing pathways regulating adult reproductive diapause in C. elegans
Source: PLoS One. 2022 Sep 16;17(9):e0274076. doi: 10.1371/journal.pone.0274076 (PMC9480990; doi:10.1371/journal.pone.0274076)
Supplement: Attachment — Submitted filename: PlosOne-ARD_ReviewerResponse_072022.pdf [file pone.0274076.s009.pdf]

Comments to the Author

1. Is the manuscript technically sound, and do the data support the conclusions?

The manuscript must describe a technically sound piece of scientific research with data that supports the conclusions. Experiments must have been conducted rigorously, with appropriate controls, replication, and sample sizes. The conclusions must be drawn appropriately based on the data presented.

Reviewer #1: Partly

Reviewer #2: Yes

---

2. Has the statistical analysis been performed appropriately and rigorously?

Reviewer #1: Yes

Reviewer #2: Yes

---

3. Have the authors made all data underlying the findings in their manuscript fully available?

The PLOS Data policy requires authors to make all data underlying the findings described in their manuscript fully available without restriction, with rare exception (please refer to the Data Availability Statement in the manuscript PDF file). The data should be provided as part of the manuscript or its supporting information, or deposited to a public repository. For example, in addition to summary statistics, the data points behind means, medians and variance measures should be available. If there are restrictions on publicly sharing data—e.g. participant privacy or use of data from a third party—those must be specified.

Reviewer #1: Yes

Reviewer #2: Yes

---

4. Is the manuscript presented in an intelligible fashion and written in standard English?

PLOS ONE does not copyedit accepted manuscripts, so the language in submitted articles must be clear, correct, and unambiguous. Any typographical or grammatical errors should be corrected at revision, so please note any specific errors here.

Reviewer #1: Yes

Reviewer #2: Yes

---

5. Review Comments to the Author

Please use the space provided to explain your answers to the questions above. You may also include additional comments for the author, including concerns about dual publication, research ethics, or publication ethics. (Please upload your review as an attachment if it exceeds 20,000 characters)

Reviewer #1: Eustice et al present a detailed genetic analysis of the metabolic requirements for a form of caloric restriction in C. elegans called adult reproductive diapause (ARD). From their data, they conclude:
1) Initiation of ARD is regulated by fatty acid metabolism, sirtuins, AMPK, and the OGlcNAc pathway
2) Maintenance of ARD is not influenced by any of the tested metabolic pathways
3) Recovery from ARD required energy sensing, stress response, insulin-like signaling, and TOR pathway function
4) Fatty acid B-oxidation regulates ARD through a ogt-1-nhr-49 pathway
5) Mutants with defects in ARD entry also exhibit changes in the levels of neutral lipids.

Overall, the experiments are extensive and make excellent use of both C. elegans and the ARD pathway to define genetic requirements for this form of caloric restriction. Experiments were performed with a high level of replication and statistical analysis of the data was robust and well done. The discussion is thorough, scholarly, and appropriate. I have some concerns related to the conclusions drawn from the genetic epistasis and the presumed functions of O-GlcNAc pathway and felt that these data could also be interpreted in ways not discussed by the authors.
Major points

1) The authors interpret the ARD entry phenotypes of the nhr-49; ogt-1, nhr-49; oga-1, and acs-2;ogt-1 double mutants in Figure 1 and 4 to mean that these genes function in the same pathway to regulate ARD entry. This is because the double mutants have phenotypes similar to the single mutants. However, these data appear to have a classic 'floor effect' problem. For example, the alternative hypothesis is that the genes operate in different pathways. In such a case, the phenotypes should be additive, leading to lower % ARD entry than either of the single mutants. However, the mutants they are analyzing already have extremely low % ARD entry (<10%) and the variability in this rate is fairly large (CV appears to be close to 100% for most). Would it even be possible to detect the alternative hypothesis that the genes function in different pathways? This has significant implications related to the model presented in Figure 6 and in the conclusions made in the discussion.

We share concerns about the genetic analysis and how we present our model in Figure 6. We agree that considering the variability and small effect size, a more conservative interpretation of the pathways is more appropriate. We have updated Figure 6 (see below) and our results and discussion in the manuscript to reflect these considerations, for example, "Because the ARD entry rate is so close to zero for *nhr-49* and *ogt-1* mutants, it was difficult to discern between an additive effect of parallel pathways or the effect of the more severe allele in a single pathway. Therefore, our epistasis experiments were unable to determine whether *nhr-49*, *oga-1*, and *ogt-1* function in the same pathway to regulate ARD entry or each have independent roles." From page 17 line 375.

Revised Figure 6

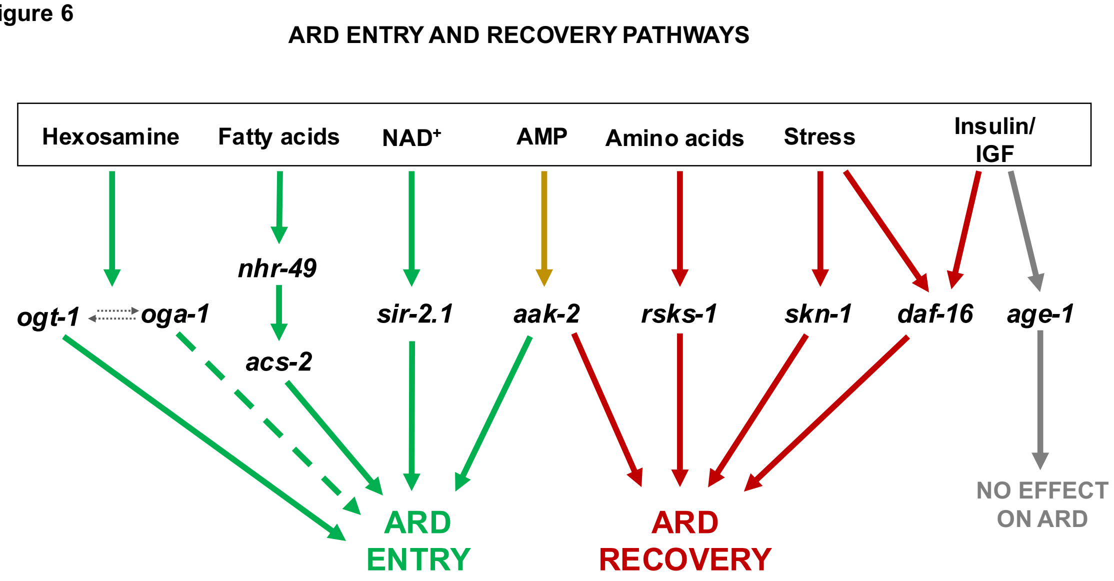

Figure 6. Model of genetic modifiers of ARD phases. Specific nutrient-sensing pathways contribute to ARD entry and exit. The initial sensing and dissemination of the signal to enter ARD (green pathways) includes genes encoding proteins involved in *O*-GlcNAc cycling (*ogt-1*, *oga-1*, with dotted arrows showing their biochemical interaction), genes that regulate fatty acid metabolism (*nhr-49*, *acs-2*) and sirtuins (*sir-2.1*). The dashed green line indicates an allele-specific effect on ARD entry. Energy sensing, mediated by the product of *aak-2*, uniquely acts in both ARD initiation and recovery (yellow pathway). The two members of the insulin/IGF signaling pathway tested in this study showed differing results with *age-1* not affecting ARD (grey pathway), while the downstream *daf-16* affected recovery (red pathway). ARD recovery additionally relies on the TOR pathway (*rsks-1*), which may also interact with *daf-16*. Stress signaling influences ARD recovery as well, with *skn-1* playing a minor role, and another possible pathway interacting with *daf-16*.

2) The authors assume that the role of ogt-1 in ARD entry is due to its catalytic addition of O-GlcNAc. However, there is ample evidence that ogt-1 also has non-catalytic roles, particularly in C. elegans. Moreover, the fact that oga-1 mutants exhibit milder phenotypes suggests there may be other aspects to the role of ogt-1 in ARD than O-GlcNAc cycling. The mutant alleles they are using eliminate both catalytic and non-catalytic activities of ogt-1. To better support their conclusion that the role of ogt-1 in ARD relates to the addition of O-GlcNAc, the authors should test catalytically inactive ogt-1 point mutants, which are publicly available.

To address this question, we have now tested the *ogt-1(dr89[K957M])* allele (previously gifted to us by Dr. Lamitina for our use in another experiment), and our results show this point mutation does not reduce ARD entry. This suggests the ARD entry phenotype does not require OGT-1 catalysis, like the multiple phenotypes that have been shown not to require catalytic activity (aldicarb hypersensitivity and osmotic stress response). These results have significantly impacted our interpretation of our data, and has been integrated into Fig 1B (see below) and the results and discussion sections. In our results section, we explain our findings as such: "Considering this and recent findings demonstrating non-

catalytic roles of OGT-1 in *C. elegans* (Giles et al. 2019, Urso et al. 2020), we tested ARD entry of worms with the catalytic-dead mutation *ogt-1(dr89[K957M])*. Interestingly, we found that this point mutant line entered ARD at similar rates to N2, demonstrating this starvation response phenotype does not require the ability of OGT-1 to transfer *O*-GlcNAc, suggesting it instead depends upon a non-catalytic role of the protein (Fig 1B)." From page 16, line 353.

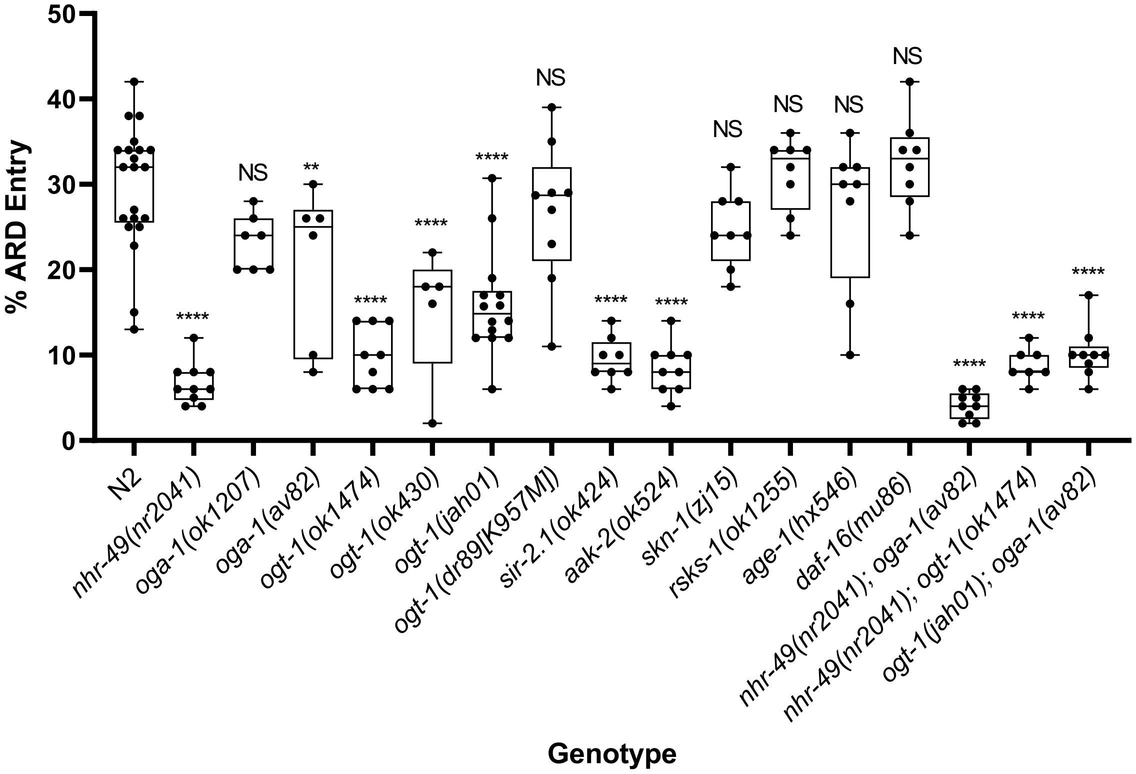

3) The authors make several qualitative comparisons related to Oil Red O staining that I do not observe. For example, they comment that the ORO staining in 'frozen' embryos is stronger in the nhr-49 and ogt-1 single mutants after 30 days of ARD as shown in Figure 5 (I presume they mean compared to N2?). To me, the mutants look either lower than or similar to N2 in these images and no quantification of the embryo ORO levels was performed. What is the evidence for this conclusion? Likewise, Figure S5 lacks an N2 control for comparison. But with the exception of the age-1 mutant, I'm not sure any of the other are obviously 'stronger' than the N2 image in Figure S5 without some type of quantification.

We agree with the reviewer that our description of these results was confusing and did not reflect the data as well as we had intended. We have revised the relevant paragraph of our results section to better explain the findings. We now explain our results with greater clarity, "ORO staining is present throughout the body of worms in fed conditions, but after 30 days in ARD, staining is greatly diminished throughout the body with the exception of the retained embryo (Fig 5A and 5B)." From page 21, line 469.

To address the point that S5 Fig did not include a control, we have included the N2 image from Fig 5 into S5 Fig, and noted in the S5 Fig legend that the N2 image is included in both.

4) Minor points

a. I'm not sure I understand the DAPI experiment in Fig S4A. Is this done in live or fixed worms? I think the authors are trying to determine if some of the mutants might have more or less cuticular permeablity to ORO that might explain the differences in staining? But if this is being done in fixed animals (as is the case for ORO), cuticular permeability should not matter since the animal is fully permeabilized.
The DAPI staining was included as a control for the permeability of the isopropanol-fixed worms. Though these animals were fixed and permeabilized, we still had concerns that the permeability may not be equivalent across genotypes. We tested several mutants which affect lipid levels and distribution, which could alter the cuticle in ways which may impact the ability of a dye such as ORO or DAPI to enter the worm's tissues. Our control showed equivalent DAPI staining, which strengthens our interpretation differences observed of the ORO results.

b. Is the term 'frozen embryo' typical for ARD field? I find this to sometimes be confusing, ie is it referring to how the sample was prepared? Would 'arrested embryo' serve the same purpose? Seems a little more precise. But I certainly understand sticking with frozen if that is the field standard.

Throughout the manuscript, we have updated our terminology for the embryos to use more descriptive language such as "retained" or "uterine". Other studies have shown the embryo is not truly arrested, so use of that term in particular is not favored by the field.

c. Line 176 – I think you mean 'Hatched L1s' and not 'Hatched L4s'?

We have corrected this typo.

Reviewer #2: The adult reproductive diapause (ARD) of C. elegans is an intriguing phenomenon from which there is still so much to learn. In this manuscript by Eustine et al, they divide the ARD in three steps: 1) entry, 2) maintenance, and 3) recovery. They explored many pathways that participate in each of these steps. They identified several regulators of ARD initiation like sir-2.1, aak-2, acs-2, ogt-1 and oga-1. They also identified skn-1, rsks-1 and daf-16 as important pathways for ARD recovery. Unexpectedly they did not find any pathway involved in ARD maintenance. They also found that the loss of the O-GlcNAc cycling enzymes converges on the fatty acid metabolic pathways that involved mitochondrial fatty acid B-oxidation. The manuscript contains a fair amount of work that has merits and contributes significantly into the understanding of ARD. However, there are few concerns. The first concern is that the authors did not review carefully all the literature about ARD. The original paper by Angelo and Van Gilst contains two findings that are not reproducible. The first one is that embryos observed during ARD are not arrested. Embryogenesis continues normally during ARD (Seidel and Kimble, 2011). Gametes production does not stop during ARD, in fact oogenesis continues slowly, and that is the main reason there are always embryos in the utero (Carranza-García and Navarro, 2019). The second one is that Angelo and Van Gilst paper showed that the caspase ced-3 is essential to maintain fertility, however later was shown that ced-3 animals are able to recover their fertility after ARD (Carranza-García and Navarro, 2019).

The insulin pathway was also originally implicated in ARD in 2019. An important contribution to the field was made in 2020 by the group of Antebi showing that the transcription factor HLH-30/TFEB promotes the morphological and physiological remodeling involved in ARD entry, survival and recovery and is not included in the discussion of this manuscript. It would be important to make the appropriate correction throughout the manuscript, and integrate all this literature to strength the paper.

We thank the reviewer for their positive comments and for directing us to these two papers. We have rewritten the introduction, results, and discussion to reflect the contribution of the findings from these studies. Our study and these two concurrently investigated many of the same pathways and genes, and as such we indicate the many ways in which our findings agree, and for where our findings diverge, we discuss possible explanations. For example, our discussion of the Antebi group's *hlh-30* paper in the context of our findings: "Gerisch et al. also identified *aak-2* as an essential player in ARD, that is both required for extended lifespan after ARD, and being a target of their newly identified ARD "master regulator" *hlh-30* (35). Four genes we identified as affecting ARD entry, *aak-2, acs-2, nhr-49,* and *oga-1*, are predicted *hlh-30* targets based on ChIP data (48), suggesting this transcription factor may act upstream of these pathways and influence ARD entry in part through transcriptional regulation of these genes." From page 25, line 566.
