# Supplementary material for: Nutrient sensing pathways regulating adult reproductive diapause in C. elegans
Source: PLoS One. 2022 Sep 16;17(9):e0274076. doi: 10.1371/journal.pone.0274076 (PMC9480990; doi:10.1371/journal.pone.0274076)
Supplement: Attachment — Submitted filename: response to reviewers80922.docx [file pone.0274076.s010.docx]

6. Review Comments to the Author

Please use the space provided to explain your answers to the questions above. You may also include additional comments for the author, including concerns about dual publication, research ethics, or publication ethics. (Please upload your review as an attachment if it exceeds 20,000 characters)

Reviewer #1: The authors have done an excellent job of responding to my previous comments and suggestions. The lack of an ADR phenotype in the catalytically dead ogt-1 allele is particularly exciting. Outstanding study - congratulations!

Thank you for the constructive comments! We are excited to share our work!

Reviewer #2: The authors have responded satisfactorily to all my concerns. The manuscript has improved considerably and will be ready for acceptance after few minor corrections.

We apologize for our oversight of these issues, all have been corrected in the text/figures.

1) Authors have changed the term “embryo arrest” in most of the manuscript but there are still a couple of places in which they still use this term. Please change it in lines 307 and 320.

We have changed “embryo arrest” to “retained embryos”.

2) Correct the line indent in line 369.

Corrected

3) Line 505: check partially spelling.

Corrected

This reviewer suggests outlining the gonad in the ARD worms in Figure 2 because it is not easy to see them.

We have outlined the gonads and updated the figure legend appropriately
